# A centrifugal microfluidic cross-flow filtration platform to separate serum from whole blood for the detection of amphiphilic biomarkers

**DOI:** 10.1038/s41598-021-84353-z

**Published:** 2021-03-05

**Authors:** Kiersten D. Lenz, Shailja Jakhar, Jing W. Chen, Aaron S. Anderson, Dylan C. Purcell, Mohammad O. Ishak, Jennifer F. Harris, Leyla E. Akhadov, Jessica Z. Kubicek-Sutherland, Pulak Nath, Harshini Mukundan

**Affiliations:** 1 Physical Chemistry and Applied Spectroscopy, Los Alamos National Laboratory, Los Alamos, NM USA; 2 Applied Modern Physics, Los Alamos National Laboratory, Los Alamos, NM USA; 3 Biosecurity and Public Health, Los Alamos National Laboratory, Los Alamos, NM USA; 4 Present Address: Sandia National Laboratory, Albuquerque, NM USA

**Keywords:** Biochemistry, Biological techniques, Biomarkers, Medical research, Engineering

## Abstract

The separation of biomarkers from blood is straightforward in most molecular biology laboratories. However, separation in resource-limited settings, allowing for the successful removal of biomarkers for diagnostic applications, is not always possible. The situation is further complicated by the need to separate hydrophobic signatures such as lipids from blood. Herein, we present a microfluidic device capable of centrifugal separation of serum from blood at the point of need with a system that is compatible with biomarkers that are both hydrophilic and hydrophobic. The cross-flow filtration device separates serum from blood as efficiently as traditional methods and retains amphiphilic biomarkers in serum for detection.

## Introduction

The separation of serum from whole blood is a necessary first step in many clinical diagnostic blood tests, since serum contains important biomarkers, whether autogenic or pathogenic, for disease diagnosis and monitoring^1–3^. Our focus is on the detection of amphiphilic bacterial biomarkers that are released into the host’s blood stream rapidly after infection^4–8^. Early detection and specific treatment of bacterial infections is necessary to help prevent the spread of antimicrobial resistance, save lives, and reduce the chances of outbreaks. Ideally, diagnosis will occur at the point of need and provide rapid intervention solutions. The development of rapid, simple, automated, safe, and inexpensive processing of blood samples can therefore benefit many diagnostic assays.

We are working towards a universal diagnostic strategy for all bacterial pathogens, including the development of novel assays to quickly detect biomarkers indicative of bacterial infection^9^. Our universal bacterial sensing strategy mimics innate immune recognition in the laboratory, facilitating diagnosis of all bacterial infection from a single clinical sample: blood. The bacterial biomarkers targeted by our assays are the same as those targeted by our human innate immune response, and are most often lipidated sugars (lipoglycans or glycolipids, such as lipopolysaccharide, lipoteichoic acid, and lipoarabinomannan). Previous work from our laboratory and others has shown that the amphiphilic biochemistry of these biomarkers causes them to be sequestered by host lipoprotein carriers, including high- and low-density lipoproteins (HDL and LDL)^4,6,7,9,10^. Because of this sequestration, it is necessary to liberate the amphiphilic biomarkers from their lipoprotein carriers in order to enable sensitive measurement. Detection can then occur via enzyme-linked immunosorbent assays (ELISA’s), waveguide-based biosensors^5,6,8,10^, or other methods^9^.

The focus of the work presented in this manuscript is the separation of serum from blood within a microfluidic device that preserves the integrity of relevant amphiphilic biomarkers present in serum. Our current sample processing method, while reliable, requires trained personnel and a multi-step laboratory procedure^11^. For use in a point-of-care setting, sample preparation should be automated in order to save time and ensure user safety, while preserving sample quality^12^. The platform must be able to process small volumes of whole blood, have a low cost of production, and offer minimal loss of sample integrity during processing. It is also beneficial to have the ability to integrate additional processing steps as dictated by the specific application.

While there have been many reported microfluidic devices that separate serum from blood, most to our knowledge have emphasized the preservation of protein and nucleic acid signatures^12–23^. Lipidic and amphiphilic biomarkers present a unique challenge, as they tend to adhere to many different surfaces, including certain plastics and dialysis membranes.

Centrifugal microfluidics is a promising area of research for the automation of a variety of processes, including biological assays and sample preparation^24,25^. The field is especially appealing for point-of-care and deployable devices, due to the fact that minimal instrumentation is needed, and the centrifugal force present is inherently effective for density-based separations^25^. This applies to our system, as the extraction of serum from blood is essentially a phase separation. We have applied the concepts of centrifugal microfluidics in order to develop a cross-flow filtration scheme for the gentle separation of serum from blood^24,26^.

**Figure 1:**
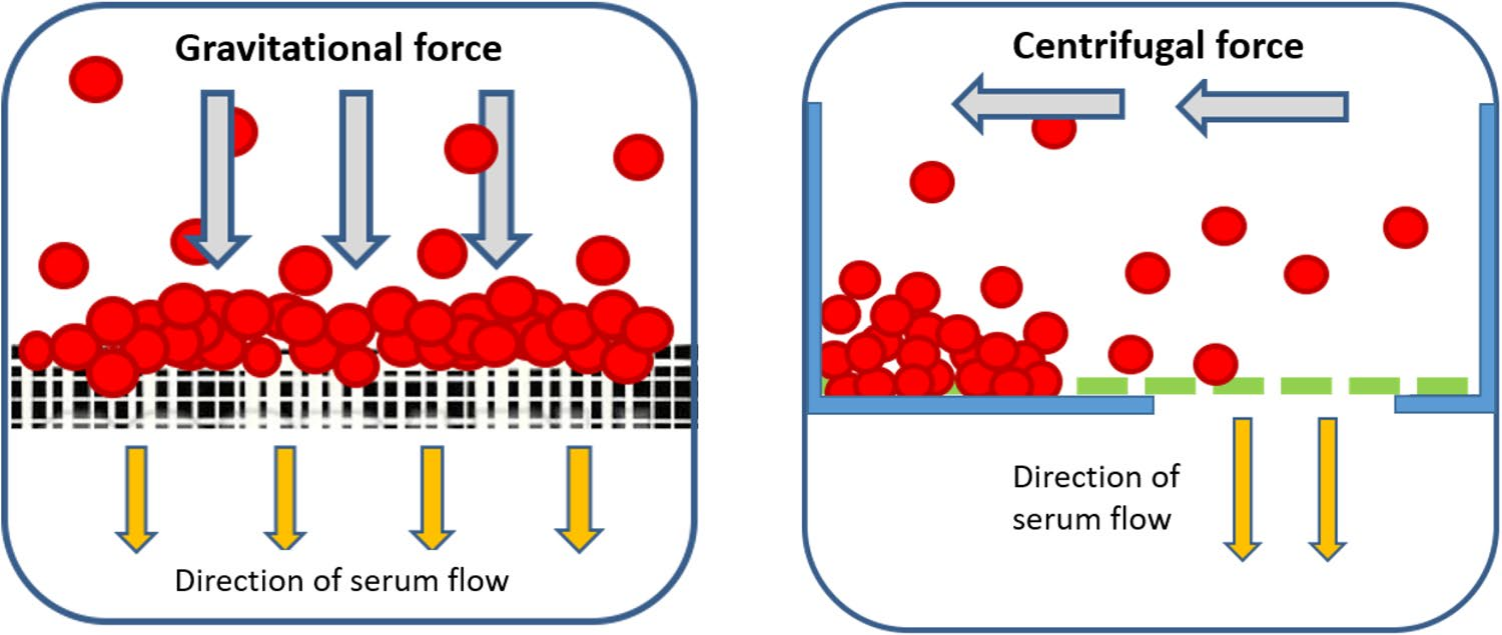
A schematic depicting the difference between the separation of serum from blood via dead-end filtration (left), which tends to cause clogging of membrane pores, and cross-flow filtration (right). Red circles represent RBCs.

During the process of cross-flow filtration, the sample passes tangentially across a filter, which is achieved via the centrifugal force acting on the platform. Components smaller than the membrane’s pores are driven through the filter as pressure increases, while larger components pass over the membrane surface^27^. In contrast, dead-end filtration can result in clumping of particles that clog the filter (Fig. 1). Cross-flow filtration decreases the chances of clogging, which in turn decreases the chances of red blood cell (RBC) lysis. Lysis of RBCs is of concern, since the release of hemoglobin (a fluorescent molecule) from the cells can interfere with fluorescence-based detection methods^28–30^.

Based on these observations, we chose to develop a cross-flow centrifugal microfluidic platform for the separation of serum from whole blood that is compatible with the biochemistry of amphiphilic biomarkers. While this is not the first time a cross-flow filtration method has been integrated into a centrifugal microfluidic chip, it is the first to our knowledge to specifically preserve amphiphilic molecules within serum^31–34^. The design was optimized to purify serum to commercial standards, as validated by performing cell counts on all samples. Materials used for the fabrication of the centrifugal platform were compatible with our sample processing criteria, as confirmed by testing samples from the device on our waveguide-based optical biosensor for the retention of amphiphilic biomarkers of interest^6,8,10,35,36^. This development greatly simplifies the sample processing requirements for our biosensing assays and facilitates the transition of such technologies to the point of need. In addition, this method can be applied to other detection techniques that require the separation and preservation of serum from whole blood, including in resource limited settings, inexpensively and rapidly.

## Working principle

**Figure 2:**
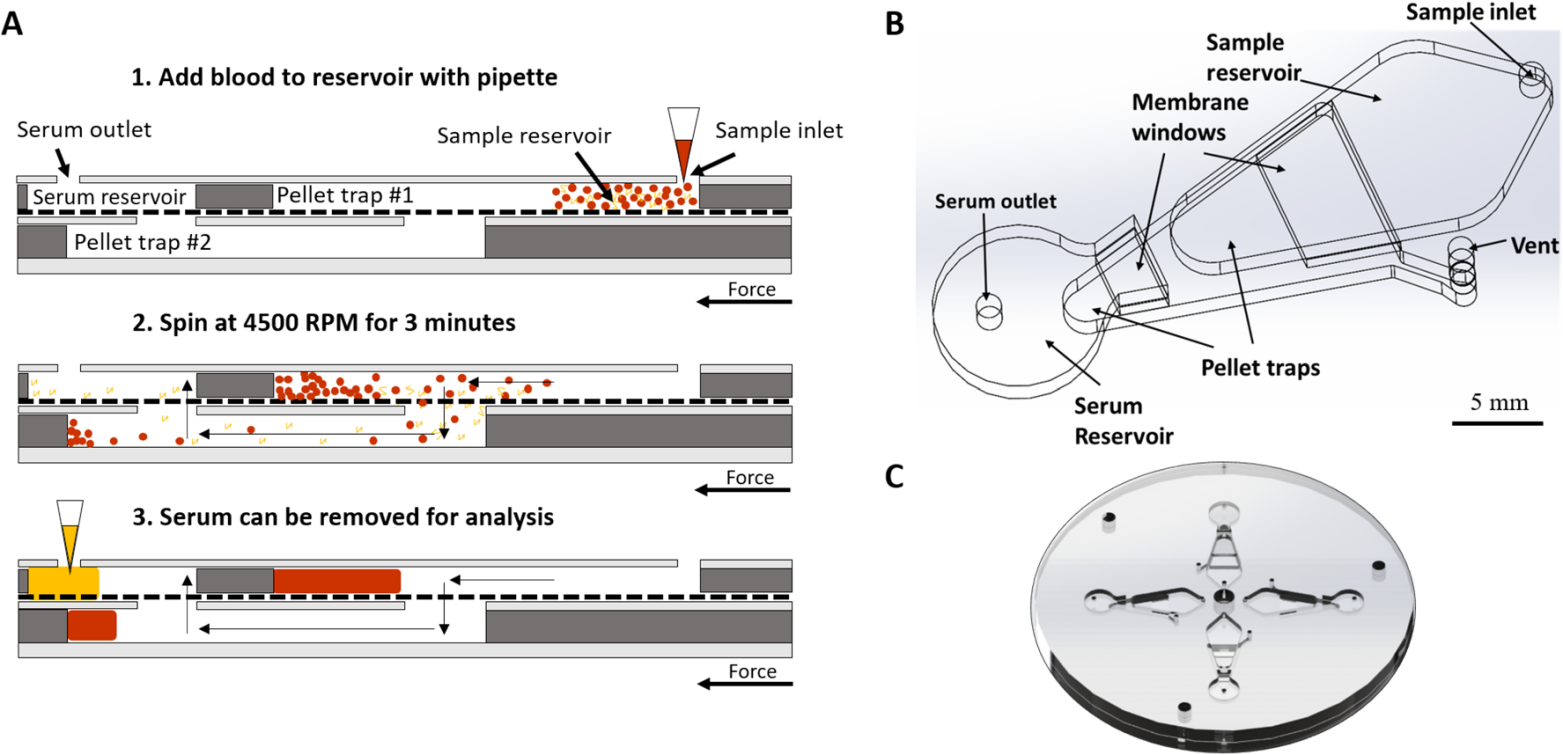
(**A**) A cross-section of one cross-flow filtration unit and separation concept; (**B**) A top-view schematic of one separation unit; (**C**) One completed disc with four separation units (diameter: 90 mm).

The device consists of multiple chambers stacked on top of each other, separated by a membrane, according to the layout in Fig. 2a,b. Four separation units are present on the centrifugal disc (Fig. 2c), enabling multiple simultaneous experiments on one chip, which could be used for various assays or replicates of the same sample. The following steps take place within each separation unit (Fig. 2a):

**Step 1:** Blood is introduced into the platform though the sample inlet. The ports are sealed, and the disc is placed on a motor to impart centrifugal force onto the sample at a given revolutions per minute (RPM). The centrifugal force causes the blood to flow away from the center of rotation. RBCs cluster toward the bottom of the sample reservoir in pellet trap #1 due to their density.

**Step 2:** As the pressure increases in the sample reservoir, serum is able to flow through the embedded membrane, while RBCs are largely prevented from flowing through due to their size. A small number of RBCs may squeeze through the pores of the membrane.

**Step 3:** Excess RBCs that went through the membrane are caught in pellet trap #2. As RBCs accumulate in pellet trap #2, the serum flows up into the serum reservoir, passing through another membrane section that serves as a secondary filtration step for RBCs. The serum is then collected through the serum outlet.

Our initial cross-flow filtration design included only one pellet trap, but we observed that some RBCs were able to get through the membrane. We realized we could easily integrate another filtration step, without changing our fabrication process, by adding another pellet trap to the design.

## Methods

### Device fabrication

The cross-flow filtration platform consists of five structural layers of plastic and one thin plastic membrane layer. The device was fabricated using a rapid prototyping method involving laser-based micropatterning and lamination. A pressure-sensitive adhesive (91,022, 3 M) was used to facilitate the lamination process. Layer schematics were drawn using SolidEdge10 2D drafting software. Alignment holes were included on all layers for assembly on a jig. The layers were cut using a CO_2_ laser cutter (M360, Universal Laser Systems) from stock cast acrylic (McMaster Carr Supply Company), polycarbonate (McMaster Carr Supply Company), and membrane sheets (Sterlitech Corporation). After cutting, the plastic layers were cleaned by bath sonication in water with dish soap for 15 min followed by a manual wash by wiping with isopropyl alcohol.

The membrane layers are Sterlitech Corporation’s track-etched hydrophilic polycarbonate with 5 µm pores. The polycarbonate membrane was coated by the manufacturer with polyvinylpyrrolidone (PVP) to ensure hydrophilicity^37^. The membrane sheets are reported by the manufacturer to be between 3 and 24 µm thick, making them delicate to work with^37,38^. In order to obtain reproducible separation by the membranes, it was important to integrate suspended membranes that are flat. We have developed a method for membrane integration, described in the Electronic Supplementary Information 1 (ESI), which consistently produces a surface that is free from visible indentations or imperfections.

### Device testing and optimization

Device functionality was verified in a series of systematic experiments that determined ideal RPM (from 3500 to 5000, tested in 500 RPM increments), time (from 2 to 5 min, tested in 1 min increments), membrane type (polycarbonate and polyester; 2, 3, and 5 µm pore sizes), and geometric design parameters (pellet trap sizes, tested in 0.5 mm height increments) for phase separations. An example of these systematic testing schemes is described in the ESI. In order to test different conditions, the disc was placed on the jig, and 90 µL of whole sheep’s blood was pipetted into each inlet hole. The inlets were designed to be the same diameter as the pipette tip in order to create a seal and prevent leakage. A one-sided custom polycarbonate tape was aligned on top of the disc to seal all ports and prevent the escape of fluids during processing. A microcentrifuge (SCI-12 High Speed Personal Micro-Centrifuge, Scilogex) was used to test different RPM and time profiles. A central hole (Fig. 2c) was cut into the microfluidic disc to fit over the rotor, and the cap from the microcentrifuge was securely fastened over the disc.

### Blood/serum separation efficiency

Serum samples from the microfluidic chip were visually inspected for hemolysis by using the CDC’s hemolysis reference palette^39^. Serum purity, defined as the percentage of cells removed from whole blood, was determined by using a TC20 Automated Cell Counter (Bio-Rad Laboratories) to compare the number of cells present in whole blood (sheep; Thermo Fisher Scientific) before separation on the microfluidic device to the number of cells present in serum after separation was complete. Serum purity was calculated using the following formula^40^: serum purity (SP) (%):

$$SP= \frac{\left(\#\;of\;cells\;in\;whole\;blood\right)-\left(\#\;of\;cells\;in\;serum\right)}{(\#\;of\;cells\;in\;whole\;blood)} \cdot 100$$

Cell counts were also performed on commercially-available sheep serum produced by ultracentrifugation (Thermo Fisher Scientific), and serum separated from whole blood in our lab by traditional benchtop methods, for comparison. Results were analyzed by a Student’s t test for significance.

### Biomarker retention

We validated the ability of our microfluidic device to retain serum biomarkers of interest by comparing the efficacy of the process against the benchtop method developed by our team. The benchtop sample processing method consists of two major steps: (1) the separation of serum from blood on a microcentrifuge; and (2) the isolation of amphiphilic biomarkers from serum using a chloroform/methanol extraction^5,6,8,10,36,41,42^. In this work, we sought to automate the first step of sample processing on a microfluidic chip, and ensure that biomarkers of interest were retained in the sample and not adsorbed to the plastic device materials.

Initial biomarker retention experiments were performed on the Los Alamos National Laboratory’s waveguide-based optical biosensor, which is used for the detection of biomarkers from Gram-negative, -positive, and -indeterminate bacteria^6,8,10,36^. The technology is described in detail elsewhere^6,8,10,35,36,41^.

The ability to retain and subsequently detect lipoarabinomannan (LAM), the virulence factor associated with *Mycobacterium tuberculosis*, was chosen as an assessment of biomarker retention. LAM is an amphiphilic biomarker, and previous work has shown that the antigen associates with HDL^7,43–46^. We have evaluated the benchtop sample processing method for the extraction of carrier-associated LAM, followed by its detection on the waveguide biosensor using a tailored method called membrane insertion^6,36,46^. Herein, we compared benchtop and microfluidic methods by measuring the sensitivity of detection of LAM in serum.

In order to test biomarker retention, whole blood was spiked with LAM to a concentration of 0.5 µM and incubated overnight at 4 °C. This concentration was chosen based on previous work that showed the linear range for LAM detection on the optical biosensor is between 0.2 and 1.0 µM^46^. The next day, serum was separated from blood using either the microfluidic device or by traditional benchtop separation, depending on the assay. For extractions using the microfluidic device, 90 µL blood was pipetted into each inlet hole, and the disc was centrifuged at 4500 RPM for 3 min. This RPM and time combination was optimized as described earlier. For traditional methods, 500 µL whole blood was pipetted into a microcentrifuge tube and centrifuged at 4500 RPM for 3 min. The serum from each method of separation was analyzed by cell counting, and sample processing was finished by benchtop methods in both cases. 120 µL of serum was mixed by pipetting with 150 µL chloroform and 300 µL methanol in low-retention microcentrifuge tubes. The mixture was spun at 5500 RPM for 1 min on a microcentrifuge, and the supernatant was discarded. The pellet containing biomarkers of interest was re-suspended in 120 µL of 1X PBS, which was injected into the flow cell of the waveguide and incubated for 45 min at room temperature. After incubation, the flow cell was washed, and the specific signal was measured on the waveguide-based optical biosensor. Complete details for the waveguide-based assays can be found in the ESI.

## Results

### Device functionality

**Figure 3:**
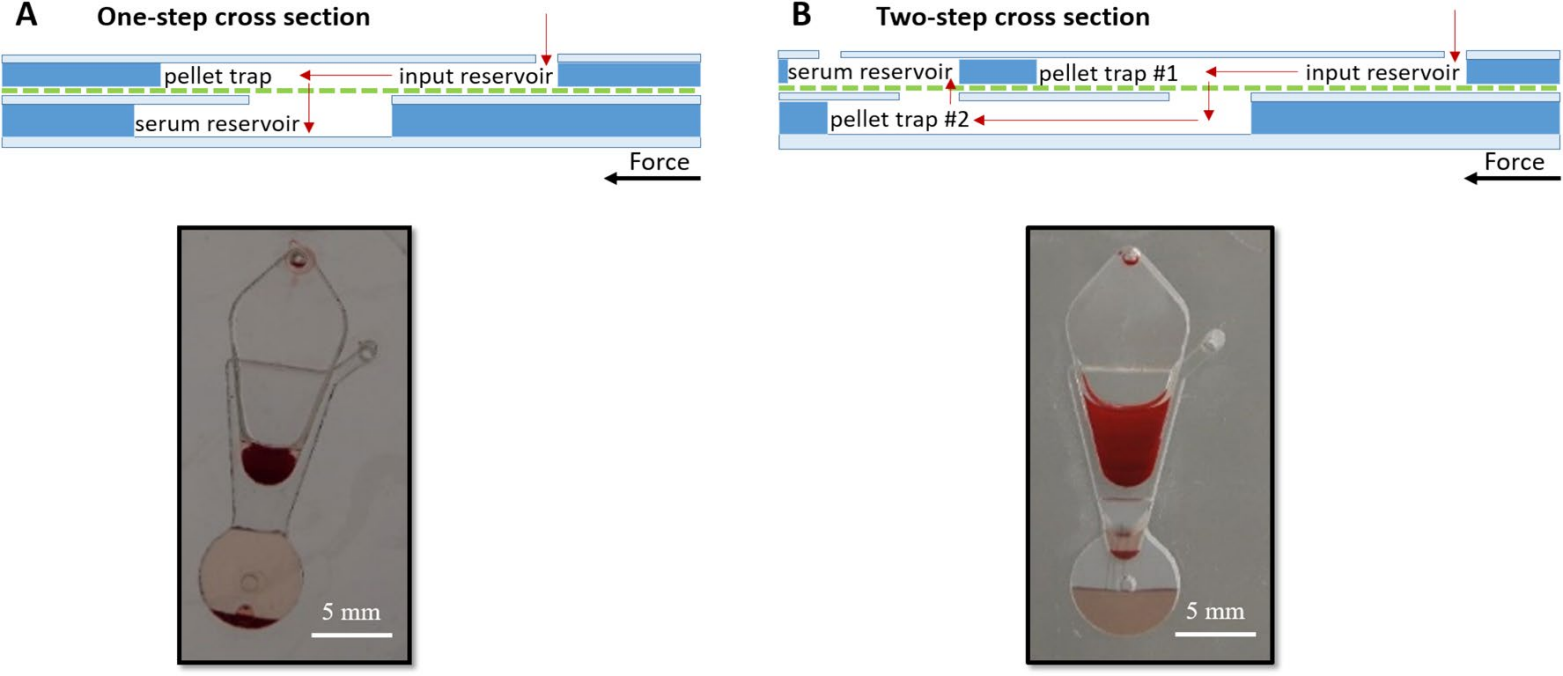
Schematics of one-step (**A**) vs. two-step (**B**) cross-flow filtration, with corresponding photos of phase separations on the microfluidic device; (**A**) the one-step filtration method was ineffective at separating a large fraction of blood cells from serum; (**B**) the two-step filtration method was successful at separating serum from blood.

We found that spinning the disc at 4500 RPM (1418 RCF) for 3 min yielded serum with the least amount of RBCs remaining in it. It is speculated that this RPM/time combination provided enough force to drive the serum through the device, but not too much force to cause hemolysis. Three minutes provided enough time for a significant amount of serum to flow through to the serum reservoir, which could then be removed for analysis. Sterlitech’s polycarbonate membrane with 5 µm pores was the most effective at filtering out RBCs. Our design includes two pellet traps for RBC collection (Fig. 2). Pellet trap heights of 5 mm and 2 mm for pellet trap #1 and #2, respectively, were found to be the most effective. A two-step filtration design was determined to be more successful at separating serum from blood when compared to a one-step method, as described in the Working Principle section. Figure 3 shows cross-sections of the one-step vs. two-step filtration designs and the resulting phase separations. An example of systematic testing parameters with results can be found in the ESI.

### Serum purity

**Figure 4:**
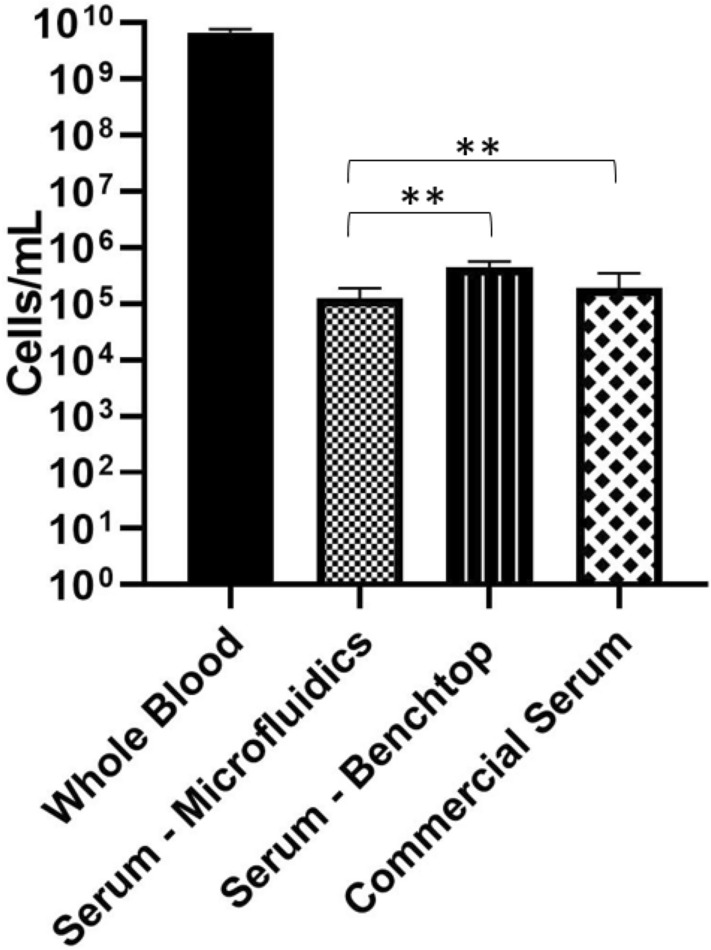
Average cell counts (n = 3) on whole blood (6.8 × 10^9^ cells/mL), serum processed on the microfluidic device (1.27 × 10^5^ cells/mL), serum processed by benchtop methods (4.45 × 10^5^ cells/mL), and commercially-available serum (1.9 × 10^5^ cells/mL). **Statistical significance.

Based on the CDC’s hemolysis reference palette, serum samples processed on the microfluidic chip contained less than 100 mg/dL of hemoglobin, which is the recommended value for serological testing^39^. We compared the RBC count of serum separated on the microfluidic device to serum separated by traditional centrifugation and to commercially-available serum. The serum processed on our microfluidic platform had a statistically significantly lower cell count (*P* = 0.0179 for microfluidics vs. benchtop; *P* = 0.0128 for microfluidics vs. commercial serum). A bar graph of cell counts for whole blood compared to different methods of separating serum is shown in Fig. 4. Serum purity was calculated for benchtop methods of separation and for the microfluidic separation. Both methods yielded a high percentage of cells removed from whole blood, greater than 99.99%.

We determined that our device is suitable for blood/serum separation, a common first step of many sample processing methods. The serum processed on the microfluidic device had a lower cell count than either the serum processed using the benchtop separation method or commercially-available serum, which indicates higher serum purity.

### Biomarker retention

**Figure 5:**
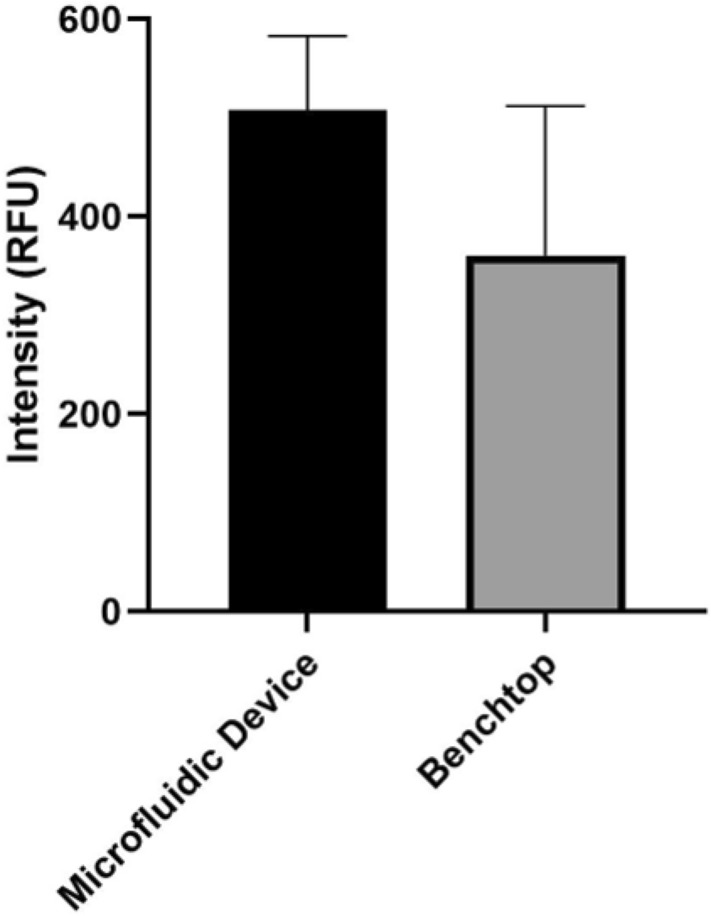
Signal intensity (n = 3) of LAM from whole sheep’s blood separated on the microfluidic device vs. benchtop methods. Higher intensity indicates higher concentration of biomarker retained in the serum sample. There was no significant difference between signals, indicating that our cross-flow filtration chip is suitable for the separation of serum from blood and subsequent detection of amphiphilic biomarkers. The fact that serum processed on the microfluidic disc yielded a similar signal intensity indicates that the microfluidic device preserves amphiphilic biomarkers present in serum as effectively as benchtop processing methods.

We validated biomarker retention for blood separated on the microfluidic chip and compared the results to our benchtop sample processing method. After serum from the microfluidic device was determined to have a significantly lower cell count (Fig. 4) than serum from the benchtop or commercially-available serum, we further validated the device by testing for biomarker retention in the sample. LAM was spiked in whole blood at 0.5 μM before separating serum from blood on the microfluidic device. Blood from the same aliquot was used for benchtop blood/serum separation in microcentrifuge tubes. After separation, the serum was analyzed on our waveguide-based optical biosensor, as described in the Experimental Section. There was no statistically significant difference between LAM levels in serum processed on the microfluidic device vs. by benchtop methods (*P* = 0.9392), indicating that the microfluidic device preserves amphiphilic biomarkers present in serum as effectively as benchtop processing methods, and the device’s materials are suitable for our application. A comparison is shown in Fig. 5.

## Discussion

We have integrated a multi-stage cross-flow filtration system into a centrifugal microfluidic platform to perform blood/serum separation. To our knowledge, this is the first cross-flow based microfluidic device capable of the separation of serum from blood with a documented ability to preserve lipidic biomarkers with the same efficiency as benchtop processing. Serum processed on our device contained fewer RBCs when compared to serum separated using benchtop methods and to commercially-available serum. The serum separated on our microfluidic chip was over 99.99% pure. There was no significant loss of signal for detection of the model biomarker of interest, LAM, when compared to benchtop methods of separation, indicating the suitability of our device for amphiphilic and lipidic signature retention.

This method of blood/serum separation offers several advantages. The platform requires only 90 µL of whole blood, which reduces invasiveness and is of importance when working with potentially dehydrated patients in remote areas of the world. This amount of blood could be collected by finger prick, which would eliminate the need for a trained phlebotomist and greatly reduce the cost per test if used at the point-of-care. It is simple to manufacture, disposable, and does not rely on pumps or valves for fluidic movement. It does not interfere with amphiphile detection as validated on our optical biosensor, nor does it require the dilution of blood. The chip itself is modular, as shown by the decision to use two pellet traps instead of one. This highlights the ease with which the design can be adapted for other phase-separation applications, without changing the manufacturing process we developed.

In this work, we have automated the first major step of our specific sample processing method, the separation of serum from blood. The platform is a promising design for the complete automation of sample processing at the point-of-care, whether for bacterial biomarkers or other lipidic signatures, which we plan to do in future work. However, our design has potential applications to human health in its current configuration. Any assays that require the separation of serum from blood could use this device to do so. For example, the common tests for cholesterol and triglycerides are typically performed directly on human serum samples. Since we have shown that our device does not interfere with the detection of lipids in serum, it could be used for this application.

The need for laboratory infrastructure for lipid handling and separation, the loss of lipidic signatures during protein processing, and the inability to process lipidic samples in field conditions remain major challenges. We plan to further develop our engineering capabilities in order to simplify sample collection, storage, processing, and measurement for lipidic signatures in biological matrices in the future.

## Supplementary Information

Supplementary Information

## Data Availability

The datasets generated during and/or analyzed used in this manuscript are available from the corresponding author on reasonable request.
