# Supplementary material for: A centrifugal microfluidic cross-flow filtration platform to separate serum from whole blood for the detection of amphiphilic biomarkers
Source: Sci Rep. 2021 Mar 5;11:5287. doi: 10.1038/s41598-021-84353-z (PMC7935985; doi:10.1038/s41598-021-84353-z)
Supplement: Supplementary file 1 — Supplementary Information [file 41598_2021_84353_MOESM1_ESM.docx]

Electronic Supplementary Information

A centrifugal microfluidic cross-flow filtration platform to separate serum from whole blood for the detection of amphiphilic biomarkers

Kiersten D. Lenz^1^, Shailja Jakhar^⸸1^, Jing W. Chen^⸸2^, Aaron S. Anderson^1^, Dylan C. Purcell^2^, Mohammad O. Ishak^3^, Jennifer F. Harris^3^, Leyla E. Akhadov^3^, Jessica Z. Kubicek-Sutherland^1^, Pulak Nath*^2^^, Harshini Mukundan*^1^

^1^ Los Alamos National Laboratory, Physical Chemistry and Applied Spectroscopy, Los Alamos NM, USA

^2^ Los Alamos National Laboratory, Applied Modern Physics, Los Alamos NM, USA

^3^ Los Alamos National Laboratory, Biosecurity and Public Health, Los Alamos NM, USA

^^^ Currently employed at Sandia National Laboratory, Albuquerque NM, USA
*Corresponding authors: pulakn.lanl@gmail.com and harshini@lanl.gov
^⸸^ These authors contributed equally to this work

**Membrane Integration**

To incorporate the membrane layer, 6-9 drops of distilled water were pushed through a syringe filter onto the clean acrylic surface of a jig. The membrane sheet with its protective top layer was gently placed down onto the clean surface. Since the membrane is hydrophilic, it is pulled onto the surface by the water through adhesion. The protective layer was gently removed without disturbing the membrane underneath. The membrane was left to dry for 5 to 10 minutes at room temperature (in Los Alamos, NM: elevation 7320 feet) to evaporate the excess water, though a slight amount of moisture underneath the membrane is desired for keeping the membrane in place. The next layer of the device was then placed on top of the membrane, glue-side down, bonding the two layers with a pressure-sensitive adhesive. The rest of the acrylic and polycarbonate layers were added using a jig and fully bonded with a hand roller as necessary. Figure S1 displays the fabrication process for membrane integration and the assembly order of bonding each layer of the device.


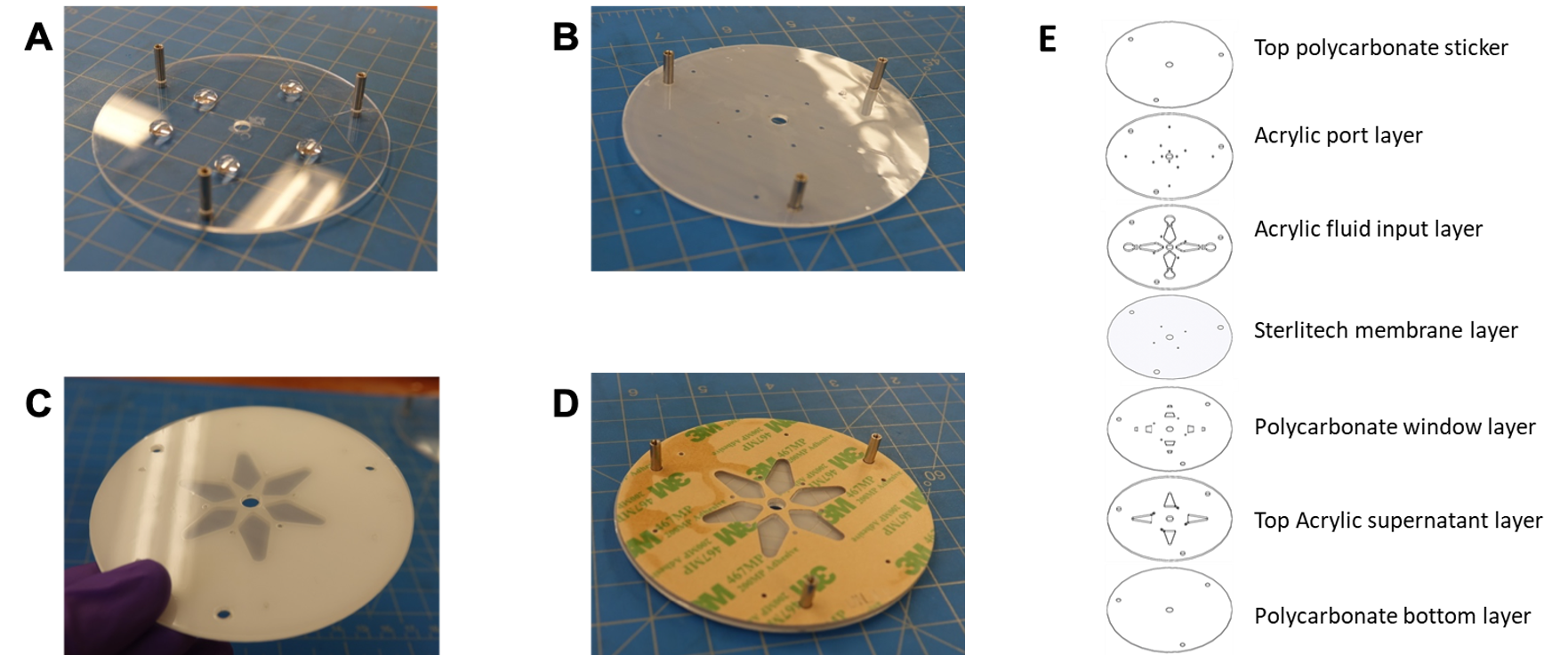


**Figure S1.** Fabrication process for consistent membrane incorporation; **A**) The jig was prepared with 5 drops of distilled water pushed through a syringe filter; **B**) The hydrophilic nature of the membrane caused it to lie flat on the jig; **C**) The next acrylic layer was bonded with a resulting smooth membrane surface; **D**) Using the jig for alignment, additional layers were added in a similar fashion; **E**) A schematic of all layers of the device in the order in which they are assembled, including a top polycarbonate sticker that is used during testing to seal the ports.

**Device Testing and Optimization**

Device functionality was verified in a series of systematic experiments that determined ideal RPM (from 3500-5000, tested in 500 RPM increments), time (from 2-5 min, tested in 1 min increments), membrane type (polycarbonate and polyester; 2, 3, and 5 µm pore sizes), and geometric design parameters (pellet trap sizes, tested in 0.5 mm height increments) for phase separations. An example of data taken for spin time and pellet trap size is presented in Table S1. Devices with pellet trap sizes from 3.5 mm to 5.0 mm were tested across spin times from 2-5 minutes, photos were taken of the results, and cells were counted in the resulting serum. These results guided decision-making on how long to spin the disc for, and what size pellet trap to use. Systematic experiments were also carried out for the other parameters described above, until a final design was optimized.

**Table S1: Device Optimization based on Spin Time and Pellet Trap Size**

|  | **Spin Time (minutes)** | | | | |
| --- | --- | --- | --- | --- | --- |
| **Pellet Trap #1 Size**  **(mm)** |  | **2** | **3** | **4** | **5** |
|  | **3.5** | **1.74x10^7^**  **cells/mL** | **2.91x10^6^**  **cells/mL** | **Too high to count** | **Too high to count** |
|  | **4.0** | **1.47x10^7^**  **cells/mL** | **3.24x10^6^**  **cells/mL** | **3.57x10^6^**  **cells/mL** | **2.05x10^6^ cells/mL** |
|  | **4.5** | **Too high to count** | **Too high to count** | **8.74x10^6^**  **cells/mL** | **1.86x10^7^ cells/mL** |
|  | **5.0** | **Too high to count** | **Too high to count** | **Too high to count** | **Too high to count** |

**Waveguide-based Assay for LAM Retention**

Planar optical waveguides with a silicon oxynitride (SiON_x_) film were prepared by nGimat (Atlanta, GA). Stock LAM (19 kDa) was procured through BEI Resources. Two LAM primary antibodies (171 and 24) were kindly provided by the Foundation for Innovative New Diagnostics and AlexaFluor 647-labeled before being prepared in a 15 nM antibody cocktail in wash buffer (0.5% BSA/1X PBS) for use on the waveguide. Dioloeyl-sn-glycero-3-phosphocholine (DOPC) and 1,2-Dioleoyl-sn-glycero-3-phasphoethanolamine-N-(cap biotinyl) (cap-biotinyl-PE) were purchased from Avanti Polar Lipids, Inc (catalog no. 850375P-25mg and 870273P-25mg). Whole sheep’s blood in Alsever’s anti-coagulant solution (2.05% dextrose, 0.8% sodium citrate, 0.0055% citric acid, 0.42% sodium chloride) was purchased from ThermoFisher Scientific, Inc (catalog no. R54044).

Waveguides and coverslips were cleaned by bath sonication for 5 minutes each in chloroform, ethanol, and distilled water. They were then dried with argon gas and cleaned by UV-ozone (UVOCS Inc.) for 40 minutes. Flow cells were assembled by bonding a silicone gasket in between clean waveguides and coverslips. The lipid bilayer was prepared from 5 mM stock DOPC and cap biotinyl-PE (both in CHCl_3_). DOPC and cap biotinyl-PE were added to a glass test tube using a syringe needle (cleaned with chloroform/ethanol 3 times each) to reach a concentration of 2mM DOPC and 1% cap biotinyl-PE. Chloroform was evaporated under argon gas, and the lipids were rehydrated in 600 µL 1X PBS for 30 minutes on a shaker plate. Lipids were then exposed to ten freeze-thaw cycles by freezing in liquid nitrogen and thawing in warm water. The fluid was then sonicated with a probe tip sonicator (Branson, 50% duty cycle) for 6 minutes (pulse 1 sec on and 1 sec off) to ensure vesicle uniformity. 70 µL of prepared lipids were pipetted into the assembled flow cell and incubated overnight at 4^o^C to encourage bilayer stabilization. Whole sheep’s blood was spiked with LAM to a concentration of 0.5 µM and incubated overnight at 4^o^C.

Each assay began by clipping the flow cell onto the custom holder and aligning the laser for coupling of light. 2 mL blocking buffer was injected through the flow cell and allowed to incubate for 1 hour at room temperature. Next, 2 mL wash buffer was injected through the flow cell and the background signal was measured (relative fluorescence units with no antigen and no antibody present). 200 µL of 15 nM antibody cocktail was injected and incubated at room temperature for 20 minutes. The flow cell was washed, and the non-specific signal, which is the interaction of the fluorescently-labelled antibody with the lipid bilayer surface in the absence of antigen, was measured. Whole blood containing LAM was separated into serum/blood using the microfluidic device or by traditional methods, depending on the assay. For the microfluidic device, 90 µL blood was pipetted into each inlet hole, and the disc was spun at 4500 RPM for 3 minutes. This RPM and time combination was optimized as described earlier. For traditional methods, 500 µL whole blood was pipetted into a microcentrifuge tube and spun at 4500 RPM for 3 minutes. The serum from each method of separation was used for cell counting, and sample processing was finished by benchtop methods. 120 µL of serum was mixed by pipetting with 150 µL chloroform and 300 µL methanol in low-retention microcentrifuge tubes. The mixture was spun at 5500 RPM for 1 minute, and the supernatant was discarded. The pellet containing amphiphiles of interest was re-suspended in 120 µL of 1X PBS, which was injected into the flow cell of the waveguide and incubated for 45 minutes at room temperature. After incubation, the flow cell was washed, and the specific signal was measured.
